# Liquid Metal Fibers with a Knitted Structure for Wearable Electronics

**DOI:** 10.3390/bios13070715

**Published:** 2023-07-07

**Authors:** Bingyi Ping, Zihang Zhang, Qiushi Liu, Minghao Li, Qingxiu Yang, Rui Guo

**Affiliations:** Department of Biomedical Engineering, Tianjin University, Tianjin 300072, China

**Keywords:** liquid metal, conductive fibers, microchannel injection, knitted structure, wearable electronics

## Abstract

Flexible conductive fibers have shown tremendous potential in diverse fields, including health monitoring, intelligent robotics, and human–machine interaction. Nevertheless, most conventional flexible conductive materials face challenges in meeting the high conductivity and stretchability requirements. In this study, we introduce a knitted structure of liquid metal conductive fibers. The knitted structure of liquid metal fiber significantly reduces the resistance variation under tension and exhibits favorable durability, as evidenced by the results of cyclic tensile testing, which indicate that their resistance only undergoes a slight increase (<3%) after 1300 cycles. Furthermore, we demonstrate the integration of these liquid metal fibers with various rigid electronic components, thereby facilitating the production of pliable LED arrays and intelligent garments for electrocardiogram (ECG) monitoring. The LED array underwent a 30 min machine wash, during which it consistently retained its normal functionality. These findings evince the devices’ robust stable circuit functionality and water resistance that remain unaffected by daily human activities. The liquid metal knitted fibers offer great promise for advancing the field of flexible conductive fibers. Their exceptional electrical and mechanical properties, combined with compatibility with existing electronic components, open new possibilities for applications in the physiological signal detection of carriers, human–machine interaction, and large-area electronic skin.

## 1. Introduction

The advancement of electronic and textile technology has led to a surge in research interest in the functionalization and intelligence of textiles [1,2,3]. Among them, the research of conductive fibers has received extensive attention. Compared with traditional metal wires, conductive fibers not only possess softness and plasticity but can also be knitted into various shapes, thereby enabling the development of diverse electronic devices [4,5]. Therefore, the conductive fibers have important application prospects in medical treatment [6], smart textiles [7], and wearable electronic devices [8,9].

Currently, the most common conductive fibers are made of inflexible metals or carbon-based materials [10,11,12]. Nevertheless, the limited tensile properties of rigid metal or carbon-based materials impede their application in wearable devices, thereby underscoring the importance of flexible conductive fibers. In recent years, liquid metal with metal-level conductivity and infinitely deformable ability has attracted widespread attention because of its potential to solve the above problems [13,14]. The extensible conductive fibers based on liquid metal have high electrical conductivity and tensile properties [15,16]. At present, the commonly used preparation methods of the stretchable conductive liquid metal fibers include template printing, microchannel injection, coat printing, and spray printing. For example, some researchers have used the template printing method to prepare a liquid metal conductive fiber with excellent electrical conductivity and flexibility [17,18,19]. In addition, researchers have used microchannel injection to prepare the conductive liquid metal fibers, which can control the fiber diameter and shape to achieve accurate regulation of fiber properties [20,21,22,23,24]. Preparation with the coating printing method involves coating the liquid metal conductive material on the surface of a fiber or another fabric to form a smart fabric of liquid metal [25,26,27]. The spray printing method involves the application of liquid metal onto fiber or fabric surfaces to create intelligent fabrics [28,29]. However, templates need to be prepared when preparing liquid metal fibers using the template printing method, and the template preparation process is relatively complicated, which increases the preparation difficulty and cost. The coat and spray printing method are relatively simple and fast preparation methods, but their functions and performance are relatively limited.

In this research, the stretchable conductive liquid metal fibers were prepared with microchannel injection. Liquid metal was injected into commercial silicone tubes to achieve a balance between electrical conductivity and softness. This method is easy to operate and has been widely used [30,31,32,33]. The knitted structure is used to further reduce the resistance change of liquid metal stretchable conductive fibers in the drawing process, meeting the conductive fibers’ diversified needs in the fields of intelligent textiles and flexible electronic devices. We also demonstrated the application prospect of stretchable conductive liquid metal fibers with a knitted structure in the field of wearable ECG monitoring. The knitted structure of the liquid metal fiber has good water resistance and durability, which provides a technical way to promote the practical application of wearable electronic devices and industrial transformation. In the future, our work will contribute to monitoring the physiological information of the carrier, human–computer interaction, and the application of large-area electronic skin.

## 2. Materials and Methods

### 2.1. Materials

Liquid metal fibers are composed of three materials, including gallium indium alloy, silicone tubes, and copper wire. Among them, the gallium indium alloy (liquid metal, 74.5% Ga and 24.5% In by weight, prepared by heating these metals at 200 °C for 2 h) is utilized as the conductive material. The silicone tube (inner diameter of 1 mm, outer diameter of 2 mm, Dow Corning 737 Neutral Cure Sealant) is employed to safeguard the liquid metal conductive structures. To encapsulate the liquid metal, a pair of copper wires are affixed to either end of the liquid metal fiber, functioning as electrodes for linking to additional circuits.

### 2.2. Preparation Method of Liquid Metal Fibers

Liquid metal fibers are made by slowly injecting liquid metal into a silicone tube at a rate of 0.1 mL/min. Firstly, the liquid metal is filled into a needle on the medical syringe and penetrated into the fiber in the hollow silicone tube. Subsequently, two copper wires with a diameter of 1.2 mm are inserted into both ends of the liquid metal fiber to facilitate connection to the analyzer and record the resistance change during the corresponding deformation. Finally, the liquid metal fibers are integrated into the cotton fabric through knitting.

### 2.3. Characterization

The mechanical properties of liquid metal fibers knitted in cotton threads with a diameter of 3 mm were measured with a high-speed extensometer (KJ-1065A; strain speed, 0.01–500 mm/min; detection precision, 0.01 N to 500 N). A digital camera (Canon 174 EOS 800D) was used to obtain the cross-sectional micro-morphology of liquid metal fibers. The measurement of electrical properties of liquid metal fiber during drawing was realized using a digital multimeter (Keithley 2002, Tektronix Inc., Beaverton, OR, USA). To mitigate the impact of the measuring electrode contact resistance, the four-wire method was employed for all electrical property evaluations.

**Figure 1:**
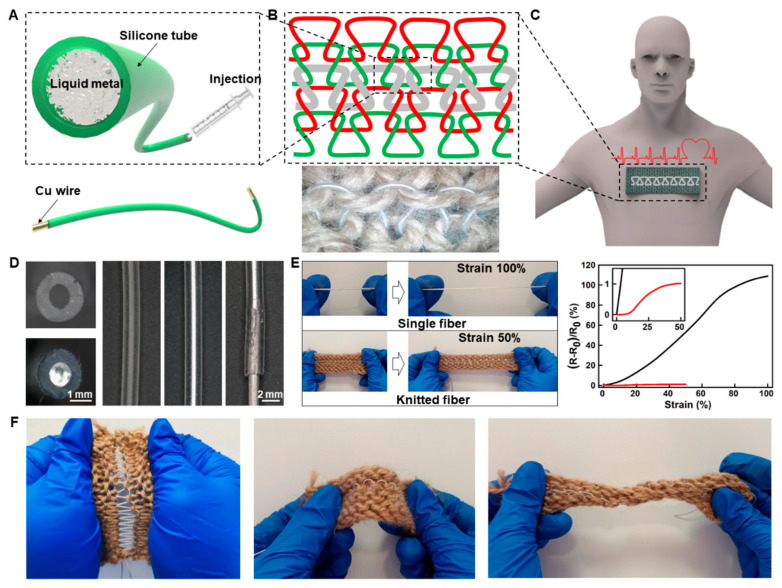
Preparation, structure, and excellent tensile stability of liquid metal fibers with a knitted structure. (**A**) The preparation and structure of a single liquid metal fiber. (**B**) The structural diagram and an image of liquid metal fiber (gray) stitched into cotton threads (red and green). (**C**) The application for wearable ECG monitoring. (**D**) The side and cross-section images of the liquid metal fiber. (**E**) Images of single and knitted liquid metal fibers at maximum stretching state, as well as their resistance change curves at different stretching states. (**F**) Images of knitted liquid metal fibers subjected to longitudinal stretching, folding, and twisting.

In this study, a single liquid metal fiber and a liquid metal fiber of knitting structure were affixed to opposite ends of the tensile table (ZQ-990L Dongguan Intelligent Precision Instrument Company, Dongguan, China) using Binder clips. The fibers were connected to equipment displayed by a step motor (J-5718 HB 4 Yueqing City Yisheng Motor Factory, Yueqing, China) end via wires to monitor resistance changes. The tensile table (ZQ-990L Dongguan Intelligent Precision Instrument Company, Dongguan, China) was designed with one fixed side and one movable side, which can be manually adjusted. A scale was incorporated to record the length of movement. At the outset, a tensile table (ZQ-990L Dongguan Intelligent Precision Instrument Company, Dongguan, China) was employed to secure a solitary fiber and a liquid metal fiber with a knitted structure at both extremities in their initial length state of 70 mm. The instrument was used to read and document the values and resistance values of this state. Subsequently, one side of the instrument was subjected to a continuous stretching of the fiber until it reached its maximum stretchable point. The alterations in fiber resistance values during this process were documented and subsequently fitted to produce Figure 1E.

The LED array underwent a machine-washing procedure utilizing a compact folding washing machine (Soseki, SOK02-A) at a speed of 240 revolutions per minute for a duration of 30 min, followed by drying with a conventional hair dryer utilizing warm air.

### 2.4. ECG Acquisition Circuit

Disposable ECG electrodes patches (commercial Ag/AgCl electrodes) connected with liquid metal fibers were directly applied to the skin as ECG electrodes. The other end of the liquid metal fiber was welded to the ECG acquisition circuit. The circuit itself was manufactured on a circuit board measuring 2.2 × 3.7 cm^2^, and included an analog front-end amplifier (BMD101, NeuroSky Electronic Technology Co., Ltd., Wuxi, China) used to collect ECG signals, as well as a Bluetooth system-on-chip (nRF52832, Nordic Semiconductor Co., Ltd., Trondheim, Norway) for signal processing and transmission.

## 3. Results and Discussions

### 3.1. Preparation of Knitted Liquid Metal Fibers

The liquid metal conductive fibers were knitted with cotton threads. Compared with other methods of transferring liquid metal patterns to textiles (such as screen printing and transfer printing), this method ensures stable fixation of the liquid metal fibers within the fabric. Furthermore, the liquid knitted metal fiber structure can significantly reduce the wire’s electrical resistance variation during fabric stretching. A liquid metal fiber is made by injecting liquid metal into a silicone tube with a syringe. The copper wires are inserted at both ends of the fiber to prevent leakage of liquid metal and to connect to other circuits (as depicted in Figure 1A). The silicone tube material utilized in this process exhibits exceptional resistance to high temperatures (250–300 °C), resistance to low temperatures (−40–60 °C), good biological safety, a tensile strength of 7.5 MPa, and an elongation capacity of up to 450%. The liquid metal used is a gallium indium alloy, which remains in a liquid state at room temperature (with a melting point of 15.4 °C), and possesses outstanding electrical conductivity (3.4 × 10^6^ S m^−1^) and low toxicity. Figure 1B shows the structural diagram and an image of liquid metal fiber (gray) stitched into cotton fibers (red and green). Among them, the diameters of liquid metal fiber and the cotton fiber are 2 mm and 4 mm, respectively. The total width of the structural unit composed of liquid metal fiber is 13 mm and the bending radius is 3 mm. This knitted fabric, featuring liquid metal conductive fiber, has potential applications in the development of smart clothing capable of monitoring human ECG signals (Figure 1C).

The determination of the diameter of the liquid metal fiber is contingent upon the specifications of the commercially available silicone tubes and their compatibility with the knitting process, specifically the minimum bending stiffness. Figure 1D shows the side and cross-section images of the liquid metal fiber. Consequently, the copper wire diameter is 1.2 mm, marginally exceeding the inner diameter of the silicone tube. Additionally, the silicone tube possesses exceptional elasticity, resulting in substantial friction when the copper wire is inserted. At a pulling speed of 30 mm/min, a copper wire located at the terminus of a 40 mm fiber experienced tensile strain of 400% and a tensile force of 9.0 N, resulting in fiber breakage and liquid metal leakage. However, the copper wire remained within the tube, thereby providing sufficient length accommodation for everyday use and ensuring electrical safety.

The maximum elongation of a single liquid metal fiber can reach 100%, while the maximum elongation of liquid metal fibers with a knitted structure is limited to 50% due to the constraint of cotton thread, as shown in the photos in Figure 1E. Here, the resistance variation rate is defined as (R − R_0_)/R_0_ × 100%. where R_0_ represents the resistance of the liquid metal resistance prior to the strain of the silicone tube, and R signifies the resistance of the liquid metal resistance after the strain of the silicone tube.

Additionally, the silicone tube cross-sectional shape changes sharply during the stretching process, resulting in a significant increase in the resistance of the liquid metal fiber (the maximum resistance variation rate: >100%), as shown by the black line in Figure 1E. In contrast, only the bending structure of the knitted liquid metal fiber is stretched during the stretching process, but the cross-sectional shape of the fiber remains unchanged. Therefore, it can prevent a significant increase in the liquid metal fiber resistance during the stretching process (the maximum resistance variation rate: <1%), as shown by the red line in Figure 1E. According to the formula of conductor resistance, the electrical resistance *R*_0_ of a regular-shaped conductor is known to follow the relationship defined by the following equation:

$$R_{0}=\rho \frac{L}{A}$$

where ρ is the resistivity of the conductor, and *L* and *A* are the length and the cross-sectional area of the conductor, respectively. In addition, the contact resistance *R*_1_ of the connection between the liquid metal and the copper wire is added to the model, as shown in Figure S1A (Supporting Information). Thus, the actual measured resistance *R* value can be calculated with the following equation:

$$R=2R_{1}+R_{0}$$

Here, we used COMSOL simulation software (Figure S1B, Supporting Information) to calculate data of the cross-sectional area and length of single fiber and knitted fiber when stretched to 50%, and from this we calculated the theoretical resistance values *R*_0_ and *R*_1_, as shown in Table S1 (Supporting Information). However, this model for simulation is an approximation and is only applicable to the elastic behavior in the small strain range. For large or nonlinear strains, this simplified model may not be applicable, and more complex strain models need to be considered or fitted using experimental data. The superior electrical stability exhibited by the knitted fiber structure allows for the creation of conductive textiles utilizing liquid metal fiber that maintain a high level of conductivity and minimal resistance fluctuation (<1%) when subjected to longitudinal stretching, folding, and twisting (Figure 1F). Consequently, the implementation of liquid metal fiber with this knitted structure presents a viable option to produce diverse wearable medical electronic devices.

### 3.2. Electrical and Mechanical Properties

**Figure 2:**
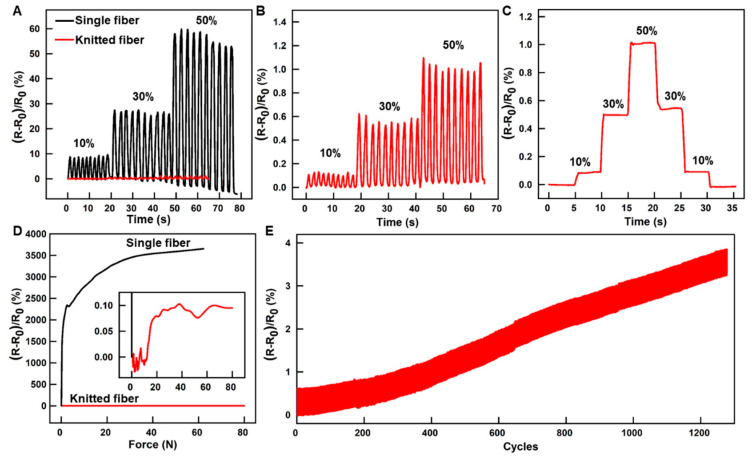
The electrical properties of single and knitted liquid metal fibers. (**A**) Resistance variation rate of single and knitted liquid metal fiber under various cyclic strains. (**B**) Resistance variation rate of knitted liquid metal fiber under various cyclic strains. (**C**) Resistance variation rate of knitted liquid metal fiber when being held at various strains. (**D**) Resistance variation rate of single and knitted liquid metal fibers under various pressures. (**E**) Resistance variation rate of knitted liquid metal fiber under cyclic tensile loading.

A battery of electrical property tests was conducted to assess the durability of the liquid metal fiber durability against daily wear. Figure 2A illustrates the stretching of single and knitted liquid metal fibers to varying degrees of strain (10%, 30% and 50%) using a tensile table, with their respective resistances recorded during the stretching process. The singular liquid metal fiber, measuring 70 mm in length, underwent a series of stretching and returning cycles. Specifically, it was subjected to a 10% strain at a rate of 7 mm/s for 10 cycles, followed by a 30% strain at a speed of 16 mm/s for 10 cycles, and finally, a 50% strain at 23 mm/s for 10 cycles. The resistance change rate of the single fiber during the experiment is depicted in the black curve of Figure 2A. The liquid metal fiber with a knitted structure, measuring 70 mm in length, underwent successive stretching to 10%, 30% and 50% strain at T = 2 s for 10 cycles. The resistance change rate of the liquid metal fiber with a knitted structure is illustrated in the red curve of Figure 2A. To demonstrate the resistance alterations of liquid metal fibers with a knitted structure under varying tensile conditions in a more comprehensible manner, the red curve in Figure 2A was magnified to produce the curve depicted in Figure 2B. The results of the tension test indicate that the rate of resistance variation of a single liquid metal fiber under distinct stretching conditions is significantly greater than that of a knitted liquid metal fiber. For example, a single liquid metal fiber exhibits a maximum resistance variation rate of 60% at 50% strain, while the knitted liquid metal fiber has a resistance variation rate of only 1.1%. Furthermore, the liquid metal fiber with a knitted structure was incrementally elongated to varying states (10%, 30% and 50%), subsequently relaxed to its initial state, and maintained at each state for a duration of 5 s. The resistance of the fiber was monitored throughout this process, as depicted in Figure 2C. It can be seen from the results that the knitted liquid metal fiber resistance remains relatively constant when subjected to elongation and relaxation at the same strain state, and exhibits favorable stability when stretched for a duration of 5 s. To assess the performance of pressure resistance of single and knitted liquid metal fiber, a range of pressures (0 to 80 N) were applied and their respective resistances were documented. Figure 2D depicts the outcomes, revealing that the resistance of the knitted liquid metal fiber remained nearly constant (<0.1%) at 80 N pressure, while the resistance of a single liquid metal fiber exhibited a variation rate of up to 3500%. The results demonstrate that the knitted liquid metal fiber can significantly improve the performance of pressure resistance, due to the protection of cotton threads. In this study, cyclic tensile tests were conducted on knitted liquid metal fiber to evaluate its resistance to daily wear, as depicted in Figure 2E. An additional experiment was performed, wherein a copper wire was pulled at a velocity of 10.5 mm/s until the tensile strain of the fiber reached 30%, with a fiber length of 70 mm. The resistance of the liquid metal was recorded at the end of each cycle, and the tests were repeated 1300 times. The cyclic tensile test results indicate that the knitted liquid metal fiber exhibits a minimal increase in resistance (<3%) after 1300 cycles, demonstrating its high durability in comparison to commercial wires. The knitted liquid metal fiber exhibits superior electrical stability and can effectively accommodate the deformations resulting from diverse human movements in everyday life.

To assess the precision of the system, we computed the error bars of the resistance rate for both the knitted liquid metal fibers and a single liquid metal fiber when subjected to varying stretching degrees (10%, 30% and 50%, with each stretch rate repeated ten times). These results are presented in Figure S2 (Supporting Information). Our findings indicate that the systematic error is negligible, the experimental data is highly stable, and the dispersion is minimal, thus lending high credibility to our results. Table S2 (Supporting Information) presents a comparison of the maximum stretchability, resistance variations and conductivity between the current work and other studies. The results indicate that the knitted liquid metal fibers exhibit comparable conductivity to other studies, while demonstrating superior electrical stability. However, the integration of cotton threads with the liquid metal fibers in the knitted structure results in reduced stretchability.

### 3.3. Interconnection with Rigid Devices

**Figure 3:**
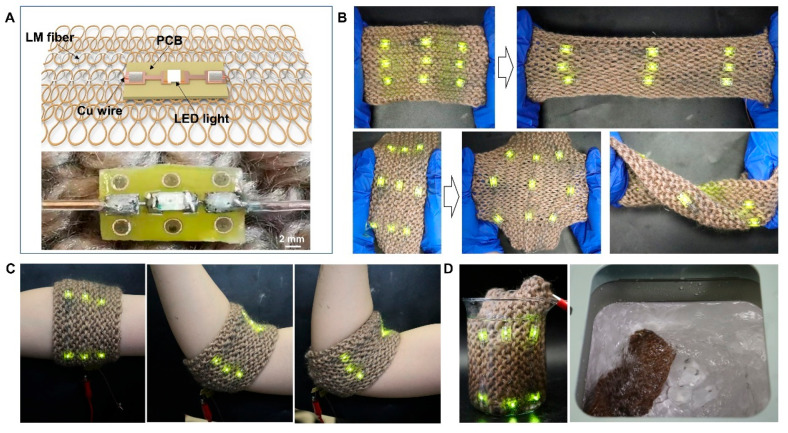
The knitted liquid metal fiber connected with LED devices. (**A**) The structural diagram and a photograph of the knitted liquid metal fiber connected with an LED device. (**B**) Photographs of the normal working LED array under various strains and twists. (**C**) The conformal LED array under different bending states of the elbow. (**D**) Photographs of the immersed in water and washed LED array.

The knitted liquid metal fibers can relate to various electronic devices to realize multi-functional wearable electronic devices. However, conventional electronic devices are usually made of rigid materials, so special fixed structures need to be designed to realize the stable connection between the liquid metal fiber and rigid devices. In this study, we illustrate the connection structure for LED lights as an example. As shown in Figure 3A, an LED light (1206) is welded on a flexible circuit board (5 mm × 10 mm), with the copper wires at both ends of the liquid metal fiber being welded to the pads of the flexible circuit board (FPCB). The flexible circuit boards are sewn onto fabric and encased in electronic epoxy for water resistance and electrical isolation. Figure 3B demonstrates that multiple LED lights are connected in series to form an LED array, which can maintain normal circuit function under various strains. In addition, the LED array can be made into irregular shapes and designs to conform to the dynamic movements and distortions of the human physique (refer to Figure 3C). Additionally, the liquid metal fiber with a knitted structure preserves the porous nature of the fabric, enabling the circuitry to be as breathable as the fabric. Throughout the washing and drying process, the LED array sustained its standard functionality. The resistance value of the liquid metal fiber exhibits a mere 3% variation, as depicted in Figure 3D. Consequently, the interconnection between the knitted liquid metal fiber and the rigid device demonstrates exceptional stability, thereby ensuring uninterrupted circuit operation during prolonged usage in everyday life.

### 3.4. Wearable ECG Monitoring System

**Figure 4:**
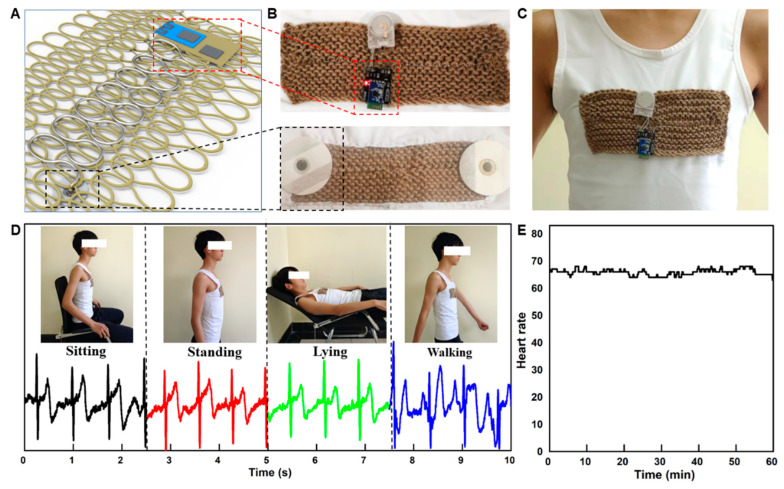
Wearable ECG monitoring system. (**A**) The overall structure of the wearable ECG monitoring system. (**B**) The ECG acquisition circuit board and commercial Ag/AgCl electrodes. (**C**) The ECG monitoring system sewn onto tight-fitting clothes. (**D**) The ECG signals of the volunteer in different states (sitting, standing, lying and walking). (**E**) The heart rate of the volunteer during sleep.

To demonstrate the practicality of this knitted liquid metal fiber for health monitoring applications, a wearable ECG monitoring system was developed to continuously monitor the ECG signals. ECG signals are an important indicator of human heart health, especially during vigorous exercise and sleep states. Figure 4A shows the overall structure of the wearable ECG monitoring system, where a knitted liquid metal fiber is used to connect the ECG electrode patches (commercial Ag/AgCl electrodes) on both sides, and the other end of the liquid metal fiber is welded to the ECG acquisition circuit board (Figure 4B). To enhance the stability of the interface between the ECG electrodes and the skin, the monitoring system was affixed to snug-fitting garments, as demonstrated in Figure 4C. Additionally, the wearable electronic device (Figure 4D) was utilized to record the ECG signals of the volunteer during various physiological states, including sitting, standing, lying and walking. Furthermore, the wearable device recorded the ECG signals of the volunteer while he was asleep for 1 h. The ECG signal measured by the electronic vest was almost identical to the ECG image of a normal adult. Figure 4E shows the heart rate curve during this period. The results show that the volunteer’s heart rates are in the range of 64–68 bpm, consistent with normal adult heart rates (60–100 bpm). These results indicate that, in the state of daily life (sitting, standing, lying and walking), the signal acquisition module on the vest is stable. Therefore, it can also measure the electrical signal of the human heart accurately. Consequently, the knitted structure of liquid metal fiber exhibits extensive potential for health monitoring during daily activities.

## 4. Conclusions

This study presents a novel technique for integrating liquid metal conductive fibers into knitted fabrics. Liquid metal fibers are made by injecting liquid metal into a silicone tube with a syringe, and copper wire is inserted at both ends of the fiber to prevent liquid metal leakage. This method can fix the liquid metal fibers stably in the fabric, and the braided structure of the liquid metal fiber can significantly reduce the wire resistance variation during stretching. Furthermore, we have devised techniques to establish a durable linkage between liquid metal fibers and inflexible apparatuses, enabling their integration with diverse electronic devices. The conductive liquid metal fiber knitted fabric holds potential for the creation of intelligent clothing capable of monitoring the electrical impulses of the human heart. In addition, the knitted fabric of liquid metal could be used to make a variety of wearable medical electronics.

Despite the favorable electrical stability of liquid metal fibers with a knitted structure under fabric stretching, this approach exhibits certain limitations. For example, the method requires hand weaving of liquid metal conductive fibers, and hence reproducibility and productivity issues with large-scale production. One possible solution is the weaving and connection of liquid metal conductive fibers using automated equipment. In addition, although wear resistance tests have been carried out in this study, long-term daily wear and cleaning may lead to damage of silicone tubes, so more durable packaging materials are needed to prevent liquid metal leakage. In the future, we believe that this knitted structure of liquid metal fiber holds immense potential in the domains of smart fabrics for human health monitoring, wearable electronic devices and intelligent robot lighting.

## Data Availability

Not applicable.

## Supplementary Materials

The following supporting information can be downloaded at: https://www.mdpi.com/article/10.3390/bios13070715/s1, Figure S1: (A) Theoretical electrical model and (B) simulation results for two fibers; Figure S2: The error bars of the resistance rate for both the knitted liquid metal fibers and a single liquid metal fiber. Table S1: The theoretical resistance values; Table S2: Summary of performances of several studies on liquid metal fibers. References [15,17,18,26] are cited in the Supplementary Materials.
