# Supplementary material for: Liquid Metal Fibers with a Knitted Structure for Wearable Electronics
Source: Biosensors (Basel). 2023 Jul 7;13(7):715. doi: 10.3390/bios13070715 (PMC10377294; doi:10.3390/bios13070715)
Supplement: Supplementary file 1 [file biosensors-13-00715-s001.zip › biosensors-2420765-supplementary.pdf]

Supporting Information

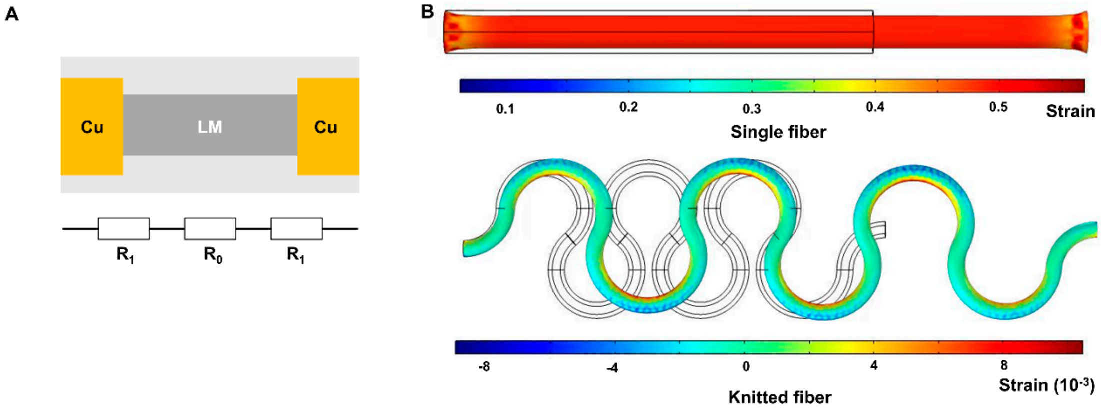

Figure S1. (**A**) Theoretical electrical model and (**B**) simulation results for two fibers.

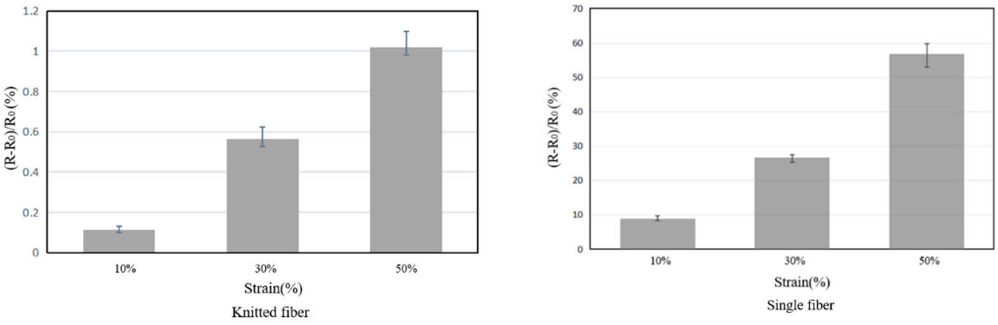

Figure S2 The error bars of the resistance rate for both the knitted liquid metal fibers and a single liquid metal fiber.

Table S1 The theoretical resistance values

	Strain	Area (A)	Length (L)	R_0	R_1
Single fiber	50%	0.66 A_0	1.5 L_0	2.25 R_0	0.68 R_0
Knitted fiber	50%	0.99 A_0	1.01 L_0	1.02 R_0	0.485 R_0

Table S2 Summary of performances of several studies on liquid metal fibers

Reference	Method and Materials	Max.stretchability	Resistance variation rate	Conductivity (S/m)
[17]	liquid metal mixed with Cu particles (Cu-EGaIn)	75%	37% at 20% strain	6×10^6
[18]	embed the patterned LMPs on the surface of polymers by casting and peeling off steps	500%	<3% with a strain of 50%	8×10^5
[26]	natural and synthetic fiber cores, and Cu-Ga-In coating	300%	5% at 100% strain	3.4×10^6
[15]	Microchannel injection	800%	> 10000%	3.4×10^6
This	Knitted Structure based on	50%	<1%	3.4×10^6

research work	microchannel injection
